# Relationship Between Angle Class I and Class II Dental Classification and Condylar Parameters Measured with a Digital Jaw Tracking System: A Cross-Sectional Study

**DOI:** 10.3390/medicina62101995

**Published:** 2026-10-15

**Authors:** Smaranda Buduru, George Cipăianu, Marius Negucioiu, Ioana Gheorghiu, Anca Ștefania Mesaroș, Răzvan Mircea Cherecheș, Carmen Biriș, Roxana Bordea, Andreea Kui, Manuela Tăut

**Affiliations:** 1 Department of Prosthetic Dentistry and Dental Materials, “Iuliu Hațieganu” University of Medicine and Pharmacy, 32 Clinicilor Street, 400006 Cluj-Napoca, Romania; dana.buduru@umfcluj.ro (S.B.); cipaianu.george@yahoo.com (G.C.);; 2 Department of Medical Education, Medical Informatics and Biostatistics, “Iuliu Hațieganu” University of Medicine and Pharmacy, 400029 Cluj-Napoca, Romania; 3 HIVE Health Innovation Center, Babeș-Bolyai University, 400347 Cluj-Napoca, Romania; 4 Faculty of Dental Medicine, “George Emil Palade” University of Medicine, Pharmacy, Science and Technology, 540139 Târgu Mureș, Romania; 5 Department of Oral Rehabilitation, “Iuliu Hațieganu” University of Medicine and Pharmacy, 400012 Cluj-Napoca, Romania

**Keywords:** sagittal condylar inclination, angle classification, digital jaw tracking, prosthodontics, digital workflow, personalized dentistry

## Abstract

*Background and Objectives*: The integration of digital jaw tracking systems into prosthodontic workflows has created new opportunities for individualized articulator programming. Sagittal condylar inclination (SCI) and Bennett angle (BA) are the key condylar guidance parameters required for this purpose, yet whether these values differ systematically across Angle dental classes remains debated. This study compared condylar guidance parameters recorded with a digital jaw tracking system between Angle Class I and Class II subjects, and assessed inter-session reliability and right-left symmetry of the recordings. *Materials and Methods*: In this prospective cross-sectional study of a convenience sample, 118 participants were included (109 Angle Class I; 9 Angle Class II; no Angle Class III participants were identified). SCI and BA were recorded with the Modjaw system (Modjaw, Villeurbanne, France) across three separate sessions during protrusion and lateral excursions of the mandible. Inter-session reliability was assessed using the intraclass correlation coefficient [ICC(3,1) and absolute-agreement ICC(A,1)] and the standard error of measurement (SEM); between-group differences with the Mann–Whitney U test; and right–left symmetry with the Wilcoxon signed-rank test (α = 0.05). *Results*: Inter-session relative reliability was high for all articular parameters (ICC(3,1) 0.953–0.991; ICC(A,1) 0.952–0.991; SEM 0.9–2.2°). No statistically significant differences in SCI or BA were identified between Class I and Class II subjects (all *p* > 0.05), although the Class II estimates were imprecise (e.g., right BA: Hodges–Lehmann estimate +4.67°, 95% CI −0.67° to +10.67°). Mean SCI in Class I ranged from 50.04° ± 9.56° to 54.05° ± 10.15°, and in Class II from 49.74° ± 13.80° to 53.04° ± 15.26°. No significant right–left asymmetry was found in either group (all *p* > 0.09). *Conclusions*: No statistically significant differences in SCI or BA were detected between the observed Angle Class I and Class II participants; however, the small and highly imbalanced Class II sample limits the precision of these estimates and does not allow conclusions regarding equivalence; no a priori sample-size calculation was performed, and only large differences (approximately 9–11° for SCI and 7–10° for BA) could have been detected. Modjaw recordings showed high inter-session relative reliability. Given the considerable inter-individual variability, patient-specific recording of condylar guidance appears preferable to estimation based on Angle class for articulator programming.

## 1. Introduction

The progressive integration of digital technologies into prosthodontic practice has fundamentally changed how condylar parameters are acquired and transferred to virtual articulators. Digital jaw tracking systems such as Modjaw (Modjaw, Villeurbanne, France) enable real-time, three-dimensional recording of mandibular kinematics and their direct fusion with intraoral scan data within fully digital prosthodontic workflows, effectively replacing conventional facebow transfers and pantographic recordings [1,2]. The two articular parameters central to this workflow are sagittal condylar inclination (SCI) and Bennett angle (BA): SCI describes the angle of condylar descent during protrusion relative to a horizontal reference plane, and BA characterizes the medial displacement of the non-working condyle during lateral mandibular movements [3]. Accurate determination of these parameters is essential for fabricating prosthodontic restorations that achieve functional harmony in both static and dynamic occlusion without interferences or premature contacts [4,5].

SCI and BA exert a stronger influence on the morphology of the posterior dentition than on the anterior, owing to their anatomical proximity to the temporomandibular joint (TMJ). Steeper SCIs produce more pronounced posterior disocclusion during protrusion and are associated with higher posterior cusps, while flatter inclinations may predispose to occlusal interferences [3]. Similarly, BA determines the degree of laterotrusive disclusion and influences cusp height and fossa depth in the transversal plane [3]. Reported mean values in adult populations range approximately from 30–60° for SCI and 7–15° for BA, depending on the recording method employed, with wide inter-individual variability documented [3,6].

Whether SCI and BA differ systematically across Angle dental classes has been investigated in several studies, with contradictory findings. Cimić et al. [6] investigated the impact of Angle classification on Bennett angle in 98 participants using a 6-degree-of-freedom ultrasound recording device and found no statistically significant differences among Class I, II/1, II/2, and Class III groups (mean BA 7.7°; one-way ANOVA, *p* = 0.789). Similarly, Pirompug et al. [7] reported no significant difference in SCI between Class I and Class II subjects (mean SCI 44.7 ± 8.8°; n = 70), nor between right and left sides, using computerized axiography. Interpreting condylar guidance values across studies is complicated by the substantial influence of the recording method on the absolute values obtained. A systematic review by Lassmann et al. [8] identified differences of up to 9° between protrusive interocclusal records and panoramic radiographs, and significantly lower condylar inclination values when wax records were used compared with pantographic recordings (*p* < 0.05), concluding that digital axiography holds potential to surpass conventional techniques in capturing functional condylar data. Regarding digital jaw tracking systems specifically, Grande et al. [9] demonstrated that optical jaw tracking produced consistent but non-negligible inter-recording variability (SD 5–7° across functional movements), underscoring that recording precision is a clinically relevant parameter when integrating jaw tracking data into CAD-based prosthodontic workflows. Evidence on the relationship between Angle classification and condylar guidance parameters recorded with digital optical jaw tracking systems remains limited.

The reliability of digital jaw tracking devices is a prerequisite for their clinical application in individualized prosthodontic workflows. Bapelle et al. [10] were among the first to systematically assess the repeatability of Modjaw recordings in 22 asymptomatic participants across two recording sessions, reporting good to excellent intraclass correlation coefficient (ICC) values for SCI at 3 mm and 5 mm and a mean SCI of 51.07° ± 9.43°. These findings were subsequently extended by Manziuc et al. [11], who evaluated Modjaw reliability across three sessions one week apart in 17 participants, obtaining excellent intrarater ICC values ranging from 0.916 to 0.993 for all SCI and BA measurements, while the ICC values for BA recorded with the Cadiax Compact 2 were comparatively lower (ICC 0.871–0.887). Notably, MODJAW produced systematically higher SCI values than the Cadiax Compact 2 by a median of 5.9° (95% CI 3.9–8.2°), highlighting that device-specific reference ranges must be considered when interpreting and comparing condylar guidance data across studies. Grande et al. [9] attributed the residual inter-recording variability of optical jaw tracking systems to both patient reproducibility of mandibular movements and operator-related factors.

The purpose of the present study was therefore to compare SCI and BA recorded with the Modjaw digital jaw tracking system between Angle Class I and Class II subjects, and secondarily to assess inter-session reliability and right–left symmetry of these measurements. The null hypothesis was that no statistically significant differences in condylar guidance parameters would be observed between Angle Class I and Class II subjects.

## 2. Materials and Methods

### 2.1. Study Design and Ethical Approval

This prospective cross-sectional observational study was conducted between March and May 2025 at the Faculty of Dental Medicine, “Iuliu Hațieganu” University of Medicine and Pharmacy, Cluj-Napoca, Romania. The study protocol was approved by the institutional Research Ethics Committee (approval no. 78/24.03.2025) and conducted in accordance with the Declaration of Helsinki. All participants provided written informed consent prior to enrollment.

### 2.2. Participants

A convenience sample of 125 adult participants was enrolled from patients presenting at the Department of Prosthetic Dentistry during the study period who met the eligibility criteria and agreed to participate. No a priori sample-size calculation was performed; the between-class comparison was therefore considered exploratory. Inclusion criteria were: age above 18 years; a complete permanent dentition (with the possible exception of third molars); absence of current signs or symptoms of temporomandibular disorders (TMDs), screened by means of the Diagnostic Criteria for Temporomandibular Disorders (DC/TMD) [12]; ability to reproducibly perform the requested mandibular movements; and willingness to attend recording sessions on consecutive days.

Exclusion criteria were: signs or symptoms of TMD according to DC/TMD screening; otolaryngological disorders; severe craniofacial skeletal anomalies; ongoing or previous orthodontic treatment; prosthetic or restorative work involving more than three tooth units; total or subtotal edentulism; history of orofacial trauma; parafunctional habits liable to alter masticatory function; neurological disorders potentially interfering with mandibular kinematics (e.g., Parkinson’s disease, previous stroke); current use of muscle relaxants or psychoactive medication; systemic diseases with joint involvement (e.g., rheumatoid arthritis); other orofacial pathologies (trigeminal neuralgia, Eagle syndrome and temporal arteritis) and inconsistent sagittal dental relationships (mixed dental classification, e.g., Angle Class I at the right canine and Angle Class II at the left canine).

### 2.3. Occlusal Classification

Full-arch intraoral scans (TRIOS 3, 3Shape A/S, Copenhagen, Denmark) were acquired for all participants at the first session and digital casts were positioned in maximum intercuspation. The sagittal inter-arch relationship was assessed independently at four reference points—right canine, left canine, right first molar, and left first molar—by an experienced prosthodontist (M.T.), using the canine and first-molar criteria of the Angle classification [3] (Table 1). Participants were assigned to a dental class only when all four reference points consistently indicated the same sagittal relationship; cases presenting a mixed classification (e.g., Angle Class I at the right canine and Angle Class II at the left canine) were excluded from the statistical analysis. Borderline relationships (deviation of less than ½ cusp from the neutral relationship) that could not be unequivocally assigned to a class were also excluded. Classification was based exclusively on the sagittal canine and first-molar relationships; overjet, overbite and incisor inclination were not recorded, so Class II Division 1 and Division 2 were not distinguished. The examiner who performed the classification also performed the kinematic recordings and was therefore not blinded to the occlusal class. Angle classification describes the dental sagittal relationship and does not necessarily reflect the skeletal pattern, which was not assessed cephalometrically in the present study.

### 2.4. Digital Jaw Tracking System

An optical jaw tracking system (Modjaw, version TWIM, Modjaw, Villeurbanne, France) was used to record the articular parameters. The device consists of a video camera connected to dedicated software running on a laptop, enabling three-dimensional recording of condylar and mandibular kinematics via a 180° camera capture beam and three sets of four motion sensors: four on a “clip” attached to the mandibular arch, four on a “headset” fixed circumferentially at the glabella and sub-occipital region, and four on a “wand” used for calibration.

### 2.5. Recording Protocol

Each participant underwent three separate recording sessions 24 h apart, all scheduled in the early morning within two hours of awakening, in order to record mandibular movements before the muscle fatigue that accumulates during the day through masticatory and other orofacial activity, and thus to standardize the physiological state of the masticatory muscles across sessions. All recordings were performed by an experienced prosthodontist (M.T.). The same device and software version (Modjaw TWIM) were used throughout, and the complete set-up, including device calibration and a new single-use fork, was repeated at each session.

**Figure 1:**
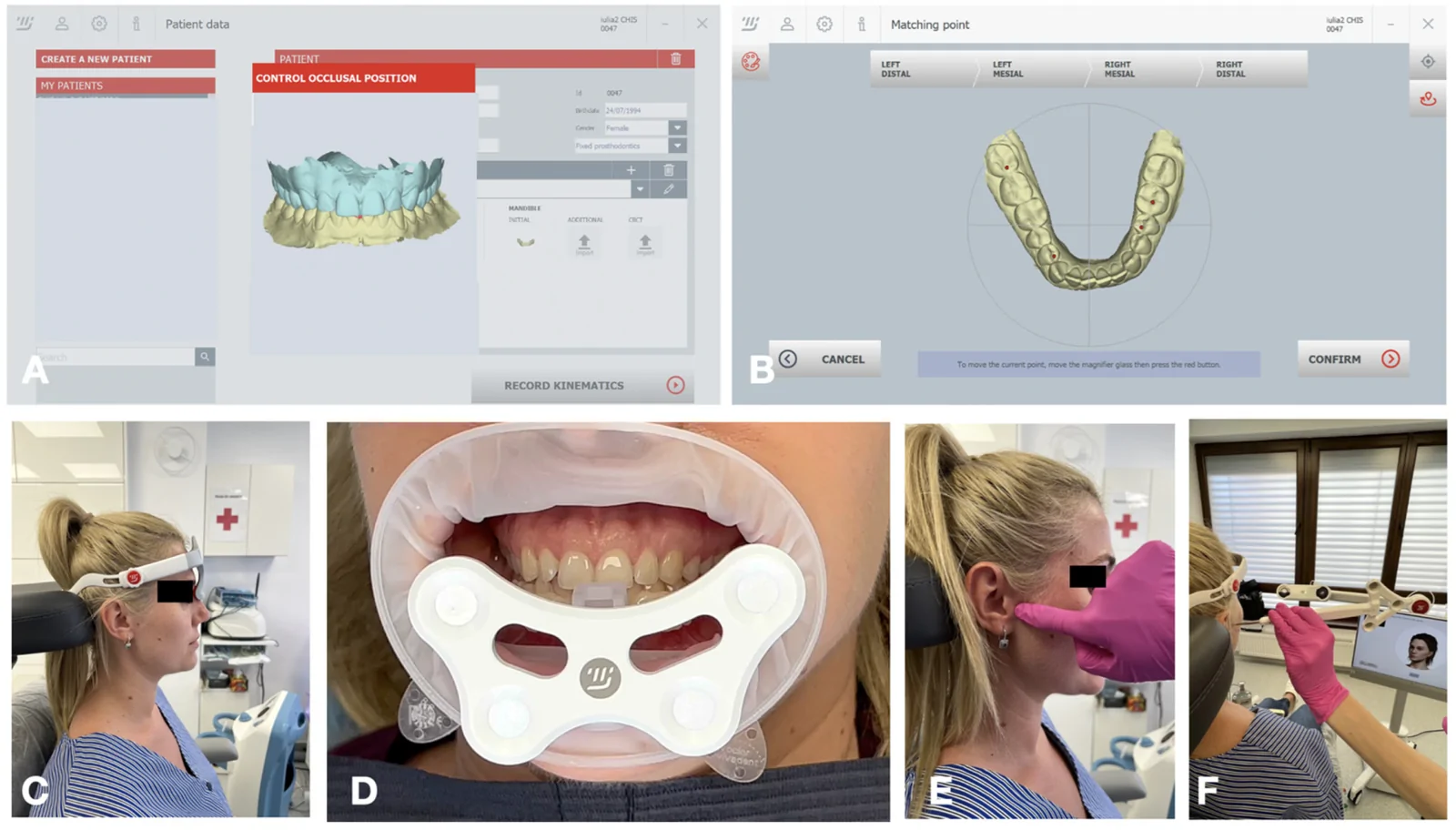
Modjaw^®^ examination steps. (**A**) Intraoral scan import; (**B**) dental point selection; (**C**) headset fitting; (**D**) clip fitting; (**E**) condylar palpation; (**F**) condylar marking.

The Modjaw examination started with the import of the intraoral scan in maximum intercuspation (STL format; Figure 1A), digital selection of four premolar fossa landmarks (Figure 1B), device calibration (Figure 1C), single-use fork attachment with self-curing resin (Protemp 4, 3M ESPE, Minnesota, USA; Figure 1D), condylar center location by palpation (Figure 1E) and digital marking (Figure 1F).

The condylar centres were located clinically by single-finger palpation during three limited opening–closing movements (up to 2 cm), marked on the skin at the first session and registered on the digital interface with the stylus of the calibration wand. These points define an arbitrary hinge axis, used only as an initial reference; the real hinge axis used for the computation of SCI and BA was determined anew by the software at each session from the centric relation recording (Section 2.6).

Recording was initiated by selecting the movement types from the dedicated menu in the following order: centric relation (CR), protrusion, right lateral excursion, and left lateral excursion. All mandibular excursions were performed in accordance with the standard condylographic convention adopted by Bapelle et al. [10] and Manziuc et al. [11]. Protrusion and lateral excursions were demonstrated and rehearsed in front of a mirror prior to recording; any incorrectly performed movement was immediately discarded and repeated. In each session, one valid protrusive movement and one valid lateral excursion to each side were recorded and analysed.

The CR recording was obtained using the single-handed mandibular manipulation technique described by Lauritzen [13], which had been practised and validated during the preliminary clinical examination. During manipulation, the patient’s head was firmly supported by the headrest and the upper and lower limbs were symmetrically positioned relative to the body’s median plane. The operator, standing to the patient’s right, placed the index finger beneath the chin and the thumb on its anterior surface, avoiding pressure on the soft tissues. Gentle, limited opening–closing movements (≤20 mm) were guided to induce pure condylar rotation without translation. Posterior mandibular displacement was deliberately avoided to prevent a defensive contraction of the lateral pterygoid muscles. CR was performed and recorded for 10 s.

### 2.6. Data Extraction

**Figure 2:**
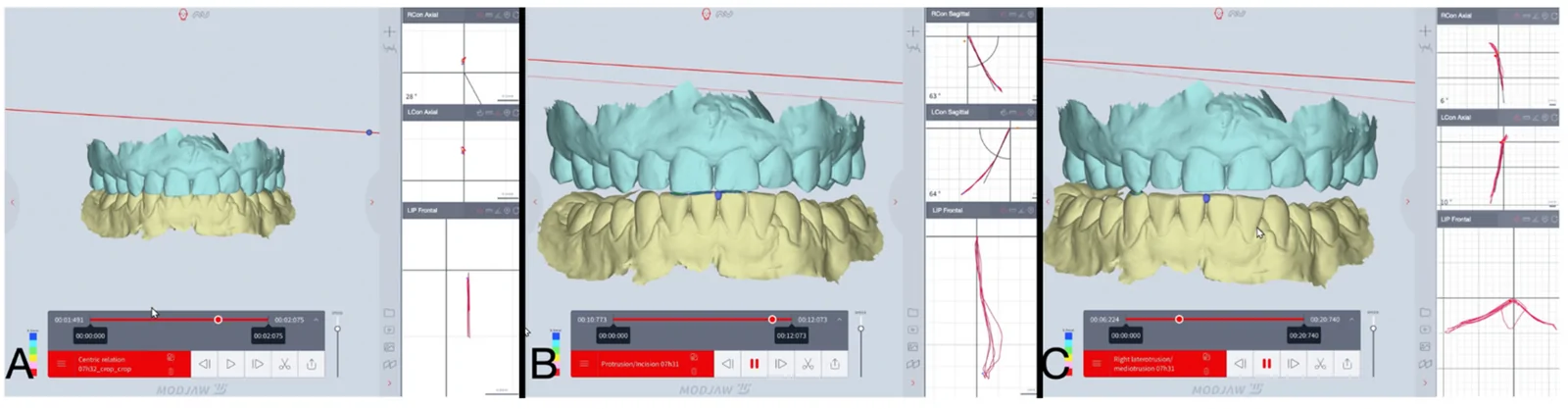
Modjaw^®^ interface. (**A**) Setting of the real condylar hinge axis; (**B**) automatic computation of sagittal condylar inclination (SCI); (**C**) automatic computation of Bennett angle (BA).

Modjaw data analysis began with validation of the CR recording. During playback, the trajectories of the right and left condyles and of the lower interincisal point were displayed in the frontal plane. When condylar displacement was confined to an area of ≤1 mm^2^ and the interincisal trajectory was rectilinear, the software automatically validated the recording and computed the pure axis of condylar rotation and the axio-orbital plane (Figure 2A).

The protrusive and lateral recordings were then assessed sequentially. During playback of each protrusive movement, the condylar and interincisal trajectories were displayed in the sagittal plane. When the condylar amplitude reached ≥5 mm, allowing extraction at both the 3 mm and 5 mm points, the software automatically computed the right and left SCI values. SCI is defined as the angle of condylar descent along the posterior slope of the articular eminence relative to a horizontal reference plane, and was extracted at 3 mm and 5 mm of protrusive condylar displacement (Figure 2B). During playback of each lateral excursion (right and left), the condylar and interincisal trajectories were displayed in the axial plane. When the condylar amplitude was ≥3 mm, the software automatically computed the right and left BA values. BA is defined as the angle of medial displacement of the contralateral condyle relative to a mid-sagittal reference plane in the axial plane, and was extracted at 3 mm of lateral condylar displacement (Figure 2C). Accordingly, BA right refers to the right condyle acting as the non-working (orbiting) condyle during the left lateral excursion performed with tooth contact, and vice versa for BA left.

Inter-session reliability was assessed on the unaveraged session-level values (three sessions per participant), as detailed in Section 2.7. For the between-group and right–left comparisons only, the values of the three sessions were averaged per parameter and per side, and the resulting participant-level means were used.

### 2.7. Statistical Analysis

Continuous variables are summarized as mean ± standard deviation (SD) and median (interquartile range, IQR). The Shapiro–Wilk test assessed distributional normality; several variables deviated from normality (*p* < 0.05). Combined with the markedly unbalanced group sizes (n = 109 vs. n = 9) and the presence of tied values, this motivated non-parametric comparisons with exact rather than asymptotic *p*-values. The Mann–Whitney U test with exact *p*-values was used for between-group comparisons (α = 0.05). For each comparison, effect sizes were computed as r = |Z|/√N, with Z derived from the normal approximation of the U statistic (interpretation: small ≥ 0.10, medium ≥ 0.30, large ≥ 0.50), and 95% confidence intervals for the median between-group difference were estimated using the Hodges–Lehmann method. Right–left symmetry was assessed using the Wilcoxon signed-rank test with exact *p*-values. Inter-session reliability was assessed using ICC(3,1): two-way mixed-effects model, single measures, consistency. ICC(3,1) was chosen because the three sessions were considered fixed, repeated occasions of the same operator and device; because a consistency ICC does not account for systematic differences between sessions, the absolute-agreement ICC(A,1) was additionally computed. Measurement error was quantified by the within-subject standard deviation, used as the standard error of measurement (SEM), the minimal detectable change (MDC95 = 1.96 × √2 × SEM), and Bland–Altman bias and 95% limits of agreement between sessions. The primary outcomes were the six articular parameters (SCI at 3 mm, SCI at 5 mm and BA, on the left and right sides) compared between Angle Class I and Class II; inter-session reliability and right–left symmetry were secondary outcomes. Because the six parameters were not combined into a single prespecified primary endpoint and are correlated, the between-group comparisons were considered exploratory, and Holm–Bonferroni-adjusted *p*-values are additionally reported for these six comparisons. The nine right–left comparisons are reported without adjustment; as none reached nominal significance, adjustment would not change their interpretation. As a sensitivity analysis for the age difference between groups, linear regression models with Angle class, age and sex as predictors (heteroscedasticity-robust HC3 standard errors) and the same models on rank-transformed outcomes were fitted for each parameter (Supplementary Table S1), and Spearman correlation coefficients between age and each articular parameter were computed in the total sample. No equivalence margin was prespecified, and no equivalence testing was performed. With the achieved group sizes (n = 109 and n = 9), a two-sided α = 0.05 and 80% power, the study could detect only standardized differences of approximately d ≈ 1.0, corresponding to approximately 9–11° for SCI and 7–10° for BA. All statistical analyses were performed using R (R Foundation for Statistical Computing, Vienna, Austria) version 4.1.2 [14]; the additional reliability, regression and sensitivity analyses were performed in Python (version 3.13) using SciPy [15], statsmodels [16] and pingouin [17].

## 3. Results

### 3.1. Sample Characteristics

**Figure 3:**
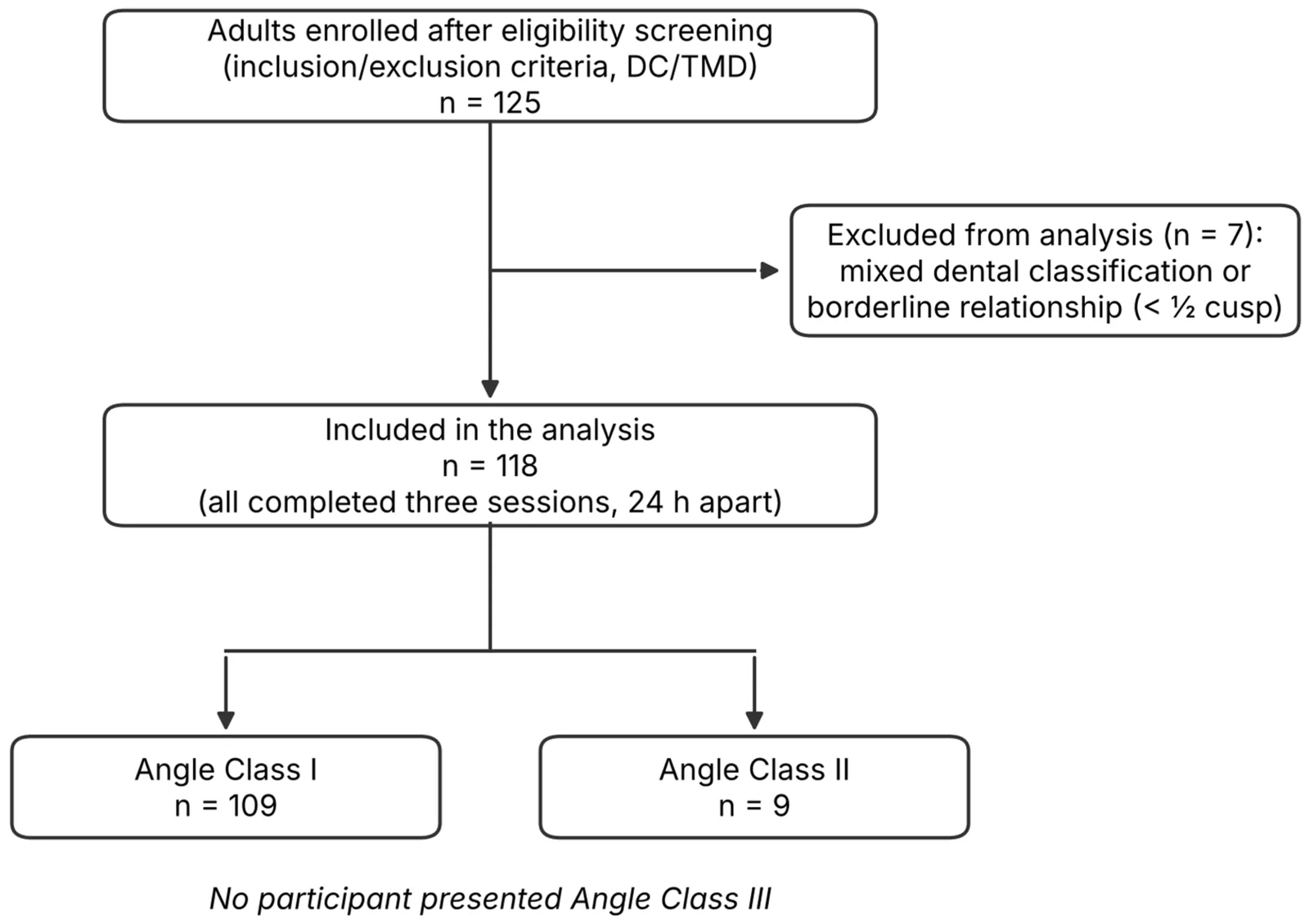
Flow diagram of participant enrolment, exclusion and analysis. DC/TMD, Diagnostic Criteria for Temporomandibular Disorders.

Of the 125 participants enrolled, 7 were excluded from the statistical analysis because the four occlusal reference points indicated inconsistent sagittal relationships across sides or tooth types (mixed dental classification) or a borderline relationship (< ½ cusp) that could not be unequivocally classified; the remaining 118 participants were included. Of these, 109 were classified as Angle Class I and 9 as Angle Class II. No participant presented with Angle Class III at either the canine or first molar reference points. The Class II group was significantly older than the Class I group (mean 41.3 ± 13.6 vs. 32.4 ± 11.8 years; Mann–Whitney U, *p* = 0.039); sex distribution did not differ between groups (*p* = 0.737). Demographic and descriptive characteristics are summarized in Table 2. All 118 participants completed the three recording sessions, with no missing data. The participant flow is shown in Figure 3.

### 3.2. Inter-Session Reliability

Inter-session ICC values for all six articular parameters were high, ranging from 0.953 to 0.991 (Table 3), indicating high inter-session relative reliability. Absolute-agreement ICCs were virtually identical (0.952–0.991), and mean differences between sessions were small for all three pairwise comparisons (S2 − S1, S3 − S1 and S3 − S2: −0.58° to +0.46°), indicating no relevant systematic shift between sessions (Table 4). The SEM ranged from 0.89° to 2.19° for SCI and from 1.16° to 1.73° for BA, and the MDC95 from 2.46° to 6.07° and from 3.21° to 4.79°, respectively. The ICC for left SCI at 5 mm was slightly lower but remained high, with wider limits of agreement (Table 4).

### 3.3. Between-Group Comparisons

**Figure 4:**
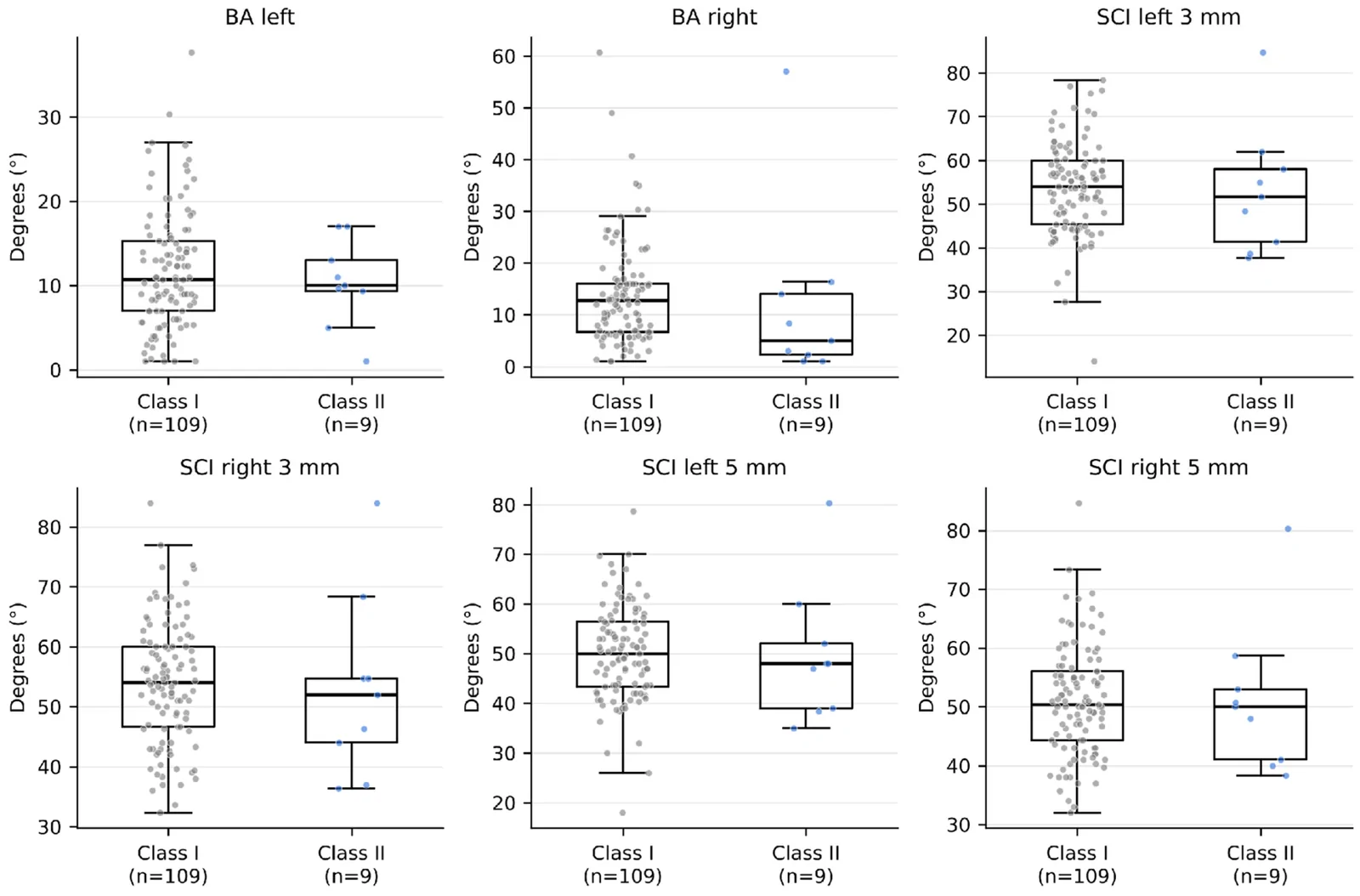
Distribution of sagittal condylar inclination (SCI) and Bennett angle (BA) by Angle class. Boxes show the median and interquartile range; whiskers, 1.5 × IQR; dots, individual participant values (mean of three sessions).

Mean SCI in Class I ranged from 50.04° ± 9.56° (left, 5 mm) to 54.05° ± 10.15° (right, 3 mm), and in Class II from 49.74° ± 13.80° (left, 5 mm) to 53.04° ± 15.26° (right, 3 mm). Mean BA in Class I was 11.75° ± 7.04° (left) and 13.79° ± 9.76° (right); in Class II, 10.33° ± 5.17° (left) and 12.00° ± 17.77° (right). None of the six articular parameter comparisons reached statistical significance (all *p* > 0.05; Table 5). Median right BA was 12.67° (IQR 6.67–16.00) in Class I and 5.00° (IQR 2.33–14.00) in Class II; the large SD in Class II was driven by a single participant with high bilateral values (right BA 57°) (Table 2; Figure 4).

Effect sizes were negligible for five of the six comparisons (r = 0.020 to 0.066), with 95% Hodges–Lehmann confidence intervals for the location shift between groups (median of all pairwise between-group differences) that all included zero. Right-sided Bennett angle showed a small effect size (r = 0.161) and a 95% confidence interval that included zero but extended to a potentially clinically relevant positive difference (Hodges–Lehmann estimate +4.67°, 95% CI [−0.67, +10.67]). After Holm–Bonferroni adjustment, all between-group *p*-values were ≥ 0.474. In the sensitivity analysis adjusted for age and sex, the Class II vs. Class I differences remained non-significant for all parameters (linear models: all *p* ≥ 0.763; rank-based models: all *p* ≥ 0.110; Supplementary Table S1); age was associated only with left BA (−0.10° per year, *p* = 0.039). In the total sample, age was not significantly correlated with any articular parameter (Spearman ρ −0.164 to +0.054; all *p* ≥ 0.076). Given the small Class II sample, these estimates are imprecise, and the absence of statistical significance should not be interpreted as evidence of equivalence.

### 3.4. Right–Left Symmetry

No statistically significant right–left difference was identified for BA, SCI at 3 mm, or SCI at 5 mm in either group or in the total sample (Table 6). Right-side BA showed a borderline non-significant trend in Class I (*p* = 0.093); all other comparisons yielded *p* ≥ 0.10. All right–left comparisons were performed on the participant-level means of the three sessions.

## 4. Discussion

This study investigated whether Angle dental classification is systematically associated with SCI and BA recorded with an optical digital jaw tracking system. The null hypothesis was that no statistically significant differences in condylar guidance parameters would be observed between Angle Class I and Class II subjects. The null hypothesis was not rejected for any of the six articular parameters analyzed. Secondary analyses confirmed high inter-session relative reliability for all parameters (ICC 0.953 to 0.991) and no statistically significant right–left asymmetry in either group. However, given the small and highly imbalanced Class II sample (n = 9) and the absence of Class III participants, these results indicate only that no statistically significant difference was detected; they do not establish equivalence between Angle classes.

The lack of a statistically significant difference in condylar guidance parameters between Angle Class I and Class II is consistent with the previous literature across a range of recording technologies. Cimić et al. [6] reported no significant differences in BA across four Angle classes in 98 participants (ultrasound six-degree-of-freedom device; mean BA 7.7°; one-way ANOVA, *p* = 0.789), and Pirompug et al. [7] found no significant differences in SCI between Class I and Class II subjects (n = 70; computerized axiography; mean SCI 44.7 ± 8.8°) nor between right and left sides. The convergence of these findings with the present optical jaw tracking data is compatible with the hypothesis that dental Angle classification is not a primary determinant of SCI or BA, although the present cross-sectional design, which allows only associations to be assessed, cannot establish this. This is anatomically plausible, as both parameters depend on the morphology of the articular eminence and the biomechanics of the temporomandibular joint, rather than on the sagittal inter-arch relationship at the level of the canines and first molars. It should also be emphasized that the present study evaluated the dental Angle relationship, not the skeletal pattern, and functional mandibular movement rather than anatomical condylar morphology. The lack of an association with Angle classification does not, however, imply that occlusal and anatomical features are unrelated to mandibular kinematics. Lassmann et al. [18], using the same Modjaw device combined with cone beam computed tomography (CBCT) in 30 subjects, reported significant correlations between the functional sagittal condylar guidance angle and both TMJ anatomical features and anterior determinants such as incisal guidance angle, overbite, and overjet. Together with the present results, this suggests that individualized recording remains preferable to any stratification based on Angle classification, and that finer anatomical and functional descriptors warrant further investigation as potential correlates of condylar guidance.

Non-significant *p*-values alone do not license a conclusion of equivalence, particularly when statistical power is limited by small subgroup sizes. The present analysis therefore reports, in addition to exact *p*-values, effect sizes (r) and 95% Hodges–Lehmann confidence intervals for the location shift between groups. For five of the six comparisons, all three quantities indicated small estimated differences: non-significant exact *p*, negligible effect size (r = 0.020 to 0.066), and confidence intervals crossing zero. Right-sided BA constituted the single exception, with a small effect size (r = 0.161) and a 95% confidence interval that included zero but extended to a potentially clinically relevant difference (median difference +4.67°, 95% CI [−0.67, +10.67], *p* = 0.079, Holm-adjusted *p* = 0.474). A small between-class difference in right-side BA cannot therefore be conclusively excluded and warrants confirmation in a larger, balanced sample. Moreover, the confidence intervals for SCI (width approximately 14–17°) remain compatible with differences of several degrees in either direction, and no equivalence margin was prespecified; formal equivalence testing (e.g., two one-sided tests) in a prospectively balanced sample would be required to establish equivalence.

The inter-session reliability observed in the present study was high, with ICC(3,1) values ranging from 0.953 to 0.991 across all six articular parameters. These values are consistent with, and in most cases exceed, those previously reported for Modjaw. Bapelle et al. [10] were among the first to systematically assess Modjaw reliability, reporting good to excellent ICC values for SCI at 3 mm and 5 mm in 22 asymptomatic participants across two recording sessions. Manziuc et al. [11] extended these observations to three sessions one week apart in 17 participants, obtaining intrarater ICC values between 0.916 and 0.993 for Modjaw measurements. The present study, with 118 participants and three sessions separated by 24 h, provides one of the largest test–retest evaluations of Modjaw reported to date and captures both biological day-to-day variability and device setup variability, a more stringent form of reliability than within-session repeatability. For comparison, Manziuc et al. [11] reported lower ICC values for BA recorded with the Cadiax Compact 2 (0.871 to 0.887), consistent with the observation that optical jaw tracking systems achieve higher reproducibility for lateral condylar movements than contact-based devices. Grande et al. [9] reported inter-recording variability of 5 to 7 degrees of standard deviation for the Cyclops optical JTS across functional movements, confirming that even optical systems retain non-negligible measurement variability attributable to both patient reproducibility and operator factors. Absolute-agreement ICCs were virtually identical to the consistency ICCs, and the SEM (0.9–2.2°) and MDC95 (2.5–6.1°) indicate that changes in SCI or BA smaller than approximately 3–6° cannot be distinguished from measurement error with this protocol. The slightly lower reproducibility of left SCI at 5 mm suggests that this parameter should be interpreted with greater caution. These reliability findings apply to the specific protocol, population, single operator and device version examined. Moreover, high ICC values demonstrate the reproducibility of repeated recordings under the study conditions, but they do not establish the accuracy or validity of the Modjaw measurements, which were not compared with an independent reference standard in the present study.

No statistically significant right–left asymmetry was identified for BA, SCI at 3 mm, or SCI at 5 mm in either group or in the total sample. The closest value to significance was observed for BA in Class I (median difference +1.00°, mean difference +2.04°, *p* = 0.093; Table 6), which remained above the pre-specified alpha threshold and is smaller than the MDC95 for BA (3.2–4.8°; Table 4). These findings extend the observation of Pirompug et al. [7], who reported symmetric SCI values in a smaller sample (n = 70), and attenuate the borderline right-side excess for SCI at 5 mm previously reported by Manziuc et al. [11] (Δ = +7.56°, *p* = 0.024) in a considerably smaller sample of 17 participants. The absence of a significant asymmetry in the present substantially larger cohort indicates that no systematic right–left difference was detected at the group level; individual patients may nevertheless show clinically relevant asymmetry, supporting patient-specific assessment when indicated.

The absolute SCI values obtained with Modjaw in the present study (approximately 50 to 54 degrees) must be interpreted in the context of a substantial device-specific offset relative to other recording modalities. Manziuc et al. [11] demonstrated that Modjaw SCI values exceed those obtained with the Cadiax Compact 2 by approximately 5.9° on average (95% CI 3.9 to 8.2). Kwon et al. [19] compared four different methods for measuring the sagittal condylar guidance angle in 20 patients and reported markedly different absolute values (protrusive interocclusal record 30.1°, panoramic radiograph 38.9°, CBCT panoramic section 35.4°, and CBCT cross section 35.3°) while observing strong Pearson correlations between methods (0.79 to 0.92). Consistent with these observations, the systematic review by Lassmann et al. [8] identified differences of up to 9° between protrusive interocclusal records and panoramic radiographs. The practical implication is that condylar guidance values are not directly interchangeable across recording modalities at the absolute level, but relative comparisons within a single-device design, as in the present study, remain internally valid. Meta-analytic and cross-study comparisons should therefore explicitly account for device-specific offsets, and clinical protocols should specify the recording modality when reporting reference values.

The lack of a detectable difference in condylar guidance parameters between Angle Class I and Class II, together with the considerable inter-individual variability of SCI and BA documented in the present sample (Table 2 and Table 5; Figure 4), may have implications for digital prosthodontic practice. Within the limits of this study, Angle Class I/II classification does not appear to be a reliable substitute for individualized measurement when pre-setting mechanical or virtual articulators in CAD/CAM-assisted restorative workflows, and average values derived from Angle class stratification may misrepresent individual patient anatomy. Consistent with the trend toward personalized prosthodontics [1,2,5,18], patient-specific digital recording of condylar guidance is preferable to estimation based on occlusal class. Optical jaw tracking systems such as Modjaw enable direct integration of individualized kinematic data with intraoral scans, within a fully digital workflow; however, the present study did not compare Modjaw with conventional facebow transfer, other recording methods or clinical outcomes, and no conclusions regarding their relative accuracy can be drawn.

Several limitations should be acknowledged. The most important is the marked group imbalance (109 Class I vs. 9 Class II) and the absence of Class III subjects, yielding limited statistical power for detecting small between-class effects. Consequently, the between-class comparison was exploratory: no a priori sample-size calculation was performed, no equivalence margin was prespecified, and the results do not establish equivalence; right-side BA in particular showed a confidence interval compatible with a clinically relevant difference. Second, participants were a convenience sample from a single university clinic, and the stringent four-point classification criterion, which excluded mixed and borderline cases, may have introduced selection bias and contributed to the small Class II group. Third, the Class II group was older than the Class I group (41.3 vs. 32.4 years). Because age-related remodelling of the temporomandibular joint, such as flattening of the articular eminence, may influence SCI and BA, age is a potential confounder of the between-class comparison; although age was not significantly correlated with any articular parameter and adjustment for age and sex did not alter the findings, the adjusted estimates are imprecise and residual confounding cannot be excluded. In addition, no Class III participants were identified. Class III is the least frequent Angle class in European populations, and the exclusion of patients with severe craniofacial skeletal anomalies or previous orthodontic treatment may have disproportionately excluded Class III individuals, introducing a potential selection bias; the findings therefore cannot be extended to Angle Class III. Fourth, Angle classification and all recordings were performed by a single, non-blinded examiner, examiner reliability of the classification was not assessed, Class II divisions were not distinguished, and the initial localization of the condyles by palpation is operator-dependent; however, SCI and BA were computed automatically by the software relative to a real hinge axis re-established at each session. Fifth, the right–left and between-class analyses were based on participant-level means; a mixed-effects model including side, session and class was not fitted because of the small Class II sample. Sixth, no minimal clinically important difference has been established for SCI or BA; the MDC95 values (Table 4) were therefore used only as a reference for differences exceeding measurement error, not as a threshold of clinical relevance. Eighth, only the reproducibility of the recordings was assessed; the accuracy of Modjaw against an independent reference standard was not evaluated. Finally, the findings apply to healthy adults with complete dentitions, no TMD, no previous orthodontic treatment and a consistent bilateral dental classification, and cannot be extrapolated to patients with TMD, orthodontic patients, patients with skeletal discrepancies or asymmetric occlusion, older adults or patients requiring complex full-arch rehabilitation. Future studies should aim for prospectively balanced enrollment across all Angle classes and subdivisions (II/1, II/2, III), incorporate covariate-adjusted analyses, and, where feasible, combine functional digital jaw tracking with anatomical imaging to elucidate the finer determinants of individual condylar guidance.

## 5. Conclusions

In this single-centre cross-sectional study, no statistically significant differences in Modjaw-recorded sagittal condylar inclination or Bennett angle were detected between the observed Angle Class I and Class II participants. The recordings demonstrated high inter-session relative reliability (ICC 0.953–0.991) and no systematic right–left differences at the group level. However, interpretation of the between-class comparisons is limited by the small and highly imbalanced Class II group and the absence of Class III participants; these findings do not establish equivalence between Angle Class I and Class II and cannot be extended to Angle Class III. High reliability indicates reproducibility under the study conditions, not measurement accuracy.

The considerable inter-individual variability observed suggests that individualized condylar guidance recordings may be preferable to reliance on Angle Class I/II classification for articulator programming.

Prospectively balanced studies including all Angle classes and integrating functional jaw tracking with anatomical imaging are warranted to further characterize the individual determinants of condylar guidance.

## Figures and Tables

**Table 1:** Angle dental classification criteria applied at four reference points.

Reference Point	Angle Class I	Angle Class II	Angle Class III
Canine (sagittal)	Upper canine occludes between lower canine and lower first premolar (neutral relationship)	Upper canine occludes on or mesial to the lower canine (½–1 cusp distalization)	Upper canine occludes with or distal to the lower first premolar (½–1 cusp mesialization)
First molar (sagittal)	Mesio-buccal cusp of upper first molar occludes in the first intercuspal groove of lower first molar (Angle’s standard)	Mesio-buccal cusp of upper first molar occludes mesial to the mesio-buccal cusp of lower first molar (½–1 cusp distalization)	Mesio-buccal cusp of upper first molar occludes distal to the centro-buccal cusp of lower first molar (½–1 cusp mesialization)

**Table 2:** Demographic and descriptive characteristics of the study sample.

Variable	Class I (n = 109)	Class II (n = 9)
Age, years (mean ± SD); median (IQR)	32.4 ± 11.8; 29.00 (23.00–39.00)	41.3 ± 13.6; 43.00 (30.00–55.00)
Sex, female/male (n)	64/45	6/3
BA left (°), mean ± SD; median (IQR)	11.75 ± 7.04; 10.67 (7.00–15.33)	10.33 ± 5.17; 10.00 (9.33–13.00)
BA right (°), mean ± SD; median (IQR)	13.79 ± 9.76; 12.67 (6.67–16.00)	12.00 ± 17.77; 5.00 (2.33–14.00)
SCI left 3 mm (°), mean ± SD; median (IQR)	53.56 ± 10.57; 54.00 (45.33–60.00)	53.04 ± 14.63; 51.67 (41.33–58.00)
SCI left 5 mm (°), mean ± SD; median (IQR)	50.04 ± 9.56; 50.00 (43.33–56.33)	49.74 ± 13.80; 48.00 (39.00–52.00)
SCI right 3 mm (°), mean ± SD; median (IQR)	54.05 ± 10.15; 54.00 (46.67–60.00)	53.04 ± 15.26; 52.00 (44.00–54.67)
SCI right 5 mm (°), mean ± SD; median (IQR)	51.02 ± 9.24; 50.33 (44.33–56.00)	51.11 ± 12.82; 50.00 (41.00–53.00)

BA, Bennett angle; SCI, sagittal condylar inclination; SD, standard deviation, IQR, interquartile range.

**Table 3:** Inter-session reliability (ICC) of Modjaw articular parameter recordings across sessions (n = 118).

Measurement	n	ICC(3,1)	95% CI	*p*
SCI right 3 mm	118	0.991	0.988–0.994	<0.001
SCI left 3 mm	118	0.990	0.986–0.993	<0.001
SCI right 5 mm	118	0.991	0.988–0.994	<0.001
SCI left 5 mm	118	0.953	0.936–0.965	<0.001
BA right	118	0.974	0.964–0.981	<0.001
BA left	118	0.972	0.963–0.980	<0.001

BA, Bennett angle; CI, confidence interval; ICC, intraclass correlation coefficient; SCI, sagittal condylar inclination. ICC(3,1): two-way mixed-effects model, single measures, consistency.

**Table 4:** Absolute agreement and measurement error of Modjaw articular parameter recordings across sessions (n = 118).

Measurement	SEM (°)	MDC95 (°)	Bias S2 − S1 (°) [95% LoA]	ICC(A,1) [95% CI]
SCI right 3 mm	0.98	2.72	−0.09 [−2.08, +1.89]	0.991 [0.99–0.99]
SCI left 3 mm	1.12	3.11	−0.33 [−3.63, +2.97]	0.989 [0.99–0.99]
SCI right 5 mm	0.89	2.46	+0.03 [−2.20, +2.27]	0.991 [0.99–0.99]
SCI left 5 mm	2.19	6.07	+0.31 [−6.71, +7.34]	0.952 [0.94–0.97]
BA right	1.73	4.79	+0.36 [−4.80, +5.51]	0.973 [0.96–0.98]
BA left	1.16	3.21	+0.14 [−2.58, +2.85]	0.972 [0.96–0.98]

BA, Bennett angle; CI, confidence interval; ICC(A,1), two-way model, absolute agreement, single measures; SEM, standard error of measurement (within-subject SD); MDC95, minimal detectable change (1.96 × √2 × SEM); LoA, Bland–Altman 95% limits of agreement between sessions 1 and 2 (session 2 − session 1); SCI, sagittal condylar inclination. The other two session pairs gave similar results (session 3 − session 1: bias −0.58° to +0.46°, widest limits for right BA, −5.20° to +6.11°; session 3 − session 2: bias −0.57° to +0.10°, widest limits for SCI left 5 mm, −7.90° to +6.77°).

**Table 5:** Between-group comparison of articular parameters (Angle Class I vs. Angle Class II).

Parameter	Class I (n = 109) Mean ± SD	Class II (n = 9) Mean ± SD	Δ (I − II)	Median Diff	95% CI (HL)	r	Interpretation	*p* *
Age, years	32.4 ± 11.8	41.3 ± 13.6	−8.9	—	—	—	—	0.039 ^†^
Sex, F/M (n)	64/45	6/3	—	—	—	—	—	0.737 ^‡^
BA left (°)	11.75 ± 7.04	10.33 ± 5.17	+1.42	+0.67°	[−3.67, +5.00]	0.020	negligible	0.812
BA right (°)	13.79 ± 9.76	12.00 ± 17.77	+1.79	+4.67°	[−0.67, +10.67]	0.161	small	0.079
SCI left 3 mm (°)	53.56 ± 10.57	53.04 ± 14.63	+0.52	+2.33°	[−6.33, +10.00]	0.058	negligible	0.527
SCI right 3 mm (°)	54.05 ± 10.15	53.04 ± 15.26	+1.01	+2.67°	[−6.00, +11.00]	0.064	negligible	0.485
SCI left 5 mm (°)	50.04 ± 9.56	49.74 ± 13.80	+0.30	+2.33°	[−5.67, +9.00]	0.066	negligible	0.485
SCI right 5 mm (°)	51.02 ± 9.24	51.11 ± 12.82	−0.09	+1.33°	[−5.67, +8.00]	0.043	negligible	0.637

BA, Bennett angle; SCI, sagittal condylar inclination; SD, standard deviation. Δ = mean Class I − mean Class II. * Mann–Whitney U test with exact *p*-values for articular parameters. ^†^ Mann–Whitney U test for age. ^‡^ Fisher exact test for sex distribution. Values represent the mean ± SD of the three recording sessions per subject and side. Δmean is the arithmetic difference between group means (descriptive). Median difference is the Hodges–Lehmann estimator, defined as the median of all pairwise between-group differences, and is the location-shift statistic associated with the Mann–Whitney U test. Effect size r was computed as |Z|/√N. Interpretation thresholds (Cohen): negligible < 0.10; small ≥ 0.10; medium ≥ 0.30; large ≥ 0.50. Mann–Whitney U statistics: BA left 512.0; BA right 663.5; SCI left 3 mm 553.0; SCI right 3 mm 559.5; SCI left 5 mm 562.0; SCI right 5 mm 537.0. Holm–Bonferroni-adjusted *p*-values for the six articular parameters: BA right 0.474; all others 1.000. Medians (IQR) are given in Table 2.

**Table 6:** Right–left symmetry of articular parameters by Angle class: Wilcoxon signed-rank test.

Parameter	Left Mean ± SD	Right Mean ± SD	Δ (R − L) Mean ± SD	*p* ^§^
Class I (n = 109)				
BA (°)	11.75 ± 7.04	13.79 ± 9.76	+2.04 ± 10.54	0.093
SCI 3 mm (°)	53.56 ± 10.57	54.05 ± 10.15	+0.49 ± 11.24	0.750
SCI 5 mm (°)	50.04 ± 9.56	51.02 ± 9.24	+0.98 ± 8.83	0.266
Class II (n = 9)				
BA (°)	10.33 ± 5.17	12.00 ± 17.77	+1.67 ± 17.86	0.570
SCI 3 mm (°)	53.04 ± 14.63	53.04 ± 15.26	0.00 ± 18.97	1.000
SCI 5 mm (°)	49.74 ± 13.80	51.11 ± 12.82	+1.37 ± 14.58	0.652
Total sample (n = 118)				
BA (°)	11.64 ± 6.91	13.65 ± 10.48	+2.01 ± 11.15	0.145
SCI 3 mm (°)	53.52 ± 10.85	53.97 ± 10.54	+0.45 ± 11.88	0.787
SCI 5 mm (°)	50.02 ± 9.87	51.03 ± 9.49	+1.01 ± 9.30	0.224

BA, Bennett angle; SCI, sagittal condylar inclination; SD, standard deviation. ^§^ Wilcoxon signed-rank test, two-sided, exact *p*-values. Tests were performed on participant-level means of the three sessions. Positive Δ indicates right-side values exceed left-side values.

## Data Availability

The raw data supporting the conclusions of this article will be made available by the authors on request.

## Supplementary Materials

The following supporting information can be downloaded at https://www.mdpi.com/article/10.3390/medicina62101995/s1, Table S1: Sensitivity analysis adjusted for age and sex.
