# Supplementary material for: Relationship Between Angle Class I and Class II Dental Classification and Condylar Parameters Measured with a Digital Jaw Tracking System: A Cross-Sectional Study
Source: Medicina (Kaunas). 2026 Oct 15;62(10):1995. doi: 10.3390/medicina62101995 (PMC13707105; doi:10.3390/medicina62101995)
Supplement: Supplementary file 1 [file medicina-62-01995-s001.zip › medicina-4568112-supplementary.pdf]

Supplementary Table S1. Sensitivity analysis adjusted for age and sex

Parameter	Class II vs I, adjusted difference (°) [95% CI]	p	Age (°/year)	p (age)	p (class, rank-based model)
BA left	-0.29 [-4.36, 3.78]	0.888	-0.103	0.039	0.827
BA right	-1.37 [-13.87, 11.13]	0.830	-0.022	0.784	0.110
SCI left 3 mm	+0.63 [-9.46, 10.72]	0.903	-0.128	0.147	0.733
SCI right 3 mm	-1.63 [-12.23, 8.96]	0.763	+0.056	0.575	0.398
SCI left 5 mm	+0.69 [-9.06, 10.45]	0.889	-0.108	0.190	0.644
SCI right 5 mm	-0.35 [-9.41, 8.72]	0.941	+0.043	0.619	0.543

Linear regression with Angle class, age and sex as predictors, robust (HC3) standard errors; rank-based model = same model on rank-transformed outcome. With n = 9 in Class II, estimates are imprecise and exploratory.
